# Bullying practices by students aged 13 to 17 years according to the National Survey of School Health (2019)

**DOI:** 10.1590/1980-549720250003

**Published:** 2025-02-10

**Authors:** Deborah Carvalho Malta, Juliana Bottoni de Souza, Évelin Angélica Herculano de Morais, Flora Vitória Serena Oliveira Baldi, Flávia Carvalho Malta de Mello, Alexandra Dias Moreira, Wanderlei Abadio de Oliveira

**Affiliations:** I Universidade Federal de Minas Gerais, School of Nursing, Department of Maternal and Child Nursing and Public Health - Belo Horizonte (MG), Brazil.; II Universidade Federal de Minas Gerais, School of Nursing - Belo Horizonte (MG), Brazil.; III Universidade de São Paulo, Ribeirão Preto School of Nursing - Ribeirão Preto (SP), Brazil.; IV Pontifícia Universidade Católica de Campinas - Campinas (SP), Brazil.

**Keywords:** Bullying, Adolescent, Tobacco, Health risk behaviors, Underage drinking

## Abstract

**Objective:**

To analyze the prevalence of bullying practices and associated factors among Brazilian adolescents, according to the National Survey of School Health 2019.

**Methods:**

This is a cross-sectional study with cluster sampling. The outcome variable, bullying practice, was assessed by the question: "In the last 30 days, have you mocked, ridiculed, teased, intimidated, or bullied any of your schoolmates to the point that they felt hurt, upset, offended, or humiliated?" (yes/no). Associations with sociodemographic characteristics, family environment, mental health, and risk behaviors were analyzed using Poisson regression with robust variance.

**Results:**

12.1% (95%CI 11.7-12.6) of adolescents reported bullying others. Positive associations were found among boys (PR 1.66; 95%CI 1.55-1.77); self-declared Black (PR 1.23; 95%CI 1.11-1.36) and brown (PR 1.1; 95%CI 1.02-1.18) adolescents; private school students (PR 1.29; 95%CI 1.21-1.37); those who felt lonely (PR 1.17; 95%CI 1.09-1.26); thought life was not worth living (PR 1.28; 95%CI 1.19-1.39); were physically assaulted by a family member (PR 1.67; 95%CI 1.55-1.79); skipped classes (PR 1.23; 95%CI 1.15-1.31); used tobacco (PR 1.34; 95%CI 1.22-1.47), alcohol (PR 1.38; 95%CI 1.28-1.50), and drugs (PR 1.17; 95%CI 1.04-1.31) regularly; and had sexual relations (PR 1.26; 95%CI 1.18-1.35). Ages 16 and 17 (PR 0.82; 95%CI 0.76-0.89) and family supervision were protective factors (PR 0.70; 95%CI 0.66-0.75).

**Conclusion:**

Bullying was more likely among boys, younger adolescents, those with family and mental health issues, and those engaged in risk behaviors. The importance of practices, such as family supervision in preventing bullying, is highlighted.

## INTRODUCTION

Bullying is a serious problem that affects the health and development of school-aged adolescents^1,2^. This phenomenon is defined as a type of violence that occurs repeatedly, based on unequal power relations between victims and aggressors, intentionally. Traditionally, bullying occurs in schools and can be physical, verbal, or psychological, resulting in consequences for everyone involved, whether victims, bullies, or bystanders^3^.

However, there are still few studies whose authors detail the experience of students identified as aggressors^3,4^. Researchers show that students who bully are also likely to have mental health problems, low empathy, a need for acceptance in the peer group, and involvement in other situations of violence^5-7^.

Furthermore, it was observed that boys practiced more all types of bullying (physical, verbal, and social) compared to girls^8^. Regarding the families of this group of students, adolescents who live in conflict contexts, with high levels of negative communication or with authoritarian parents, are more likely to display bullying behavior at school^9,10^. Being an aggressor has also been associated with low maternal education^11,12^ and unfavorable sociodemographic conditions^13^.

This scenario demonstrates the complexity of bullying practices by adolescents and highlights the importance of considering the particularities of the role played by students in these situations. Hence, it becomes possible and important to analyze the scenario of bullying practice considering individual and contextual factors associated with this practice. Thus, our objective was to analyze the prevalence of bullying and associated factors among Brazilian adolescents according to data from the National School Health Survey (*Pesquisa Nacional de Saúde do Escolar* - PeNSE) 2019.

## METHODS

This is a cross-sectional and analytical epidemiological study with data from PeNSE 2019, a national survey carried out by the Brazilian Institute of Geography and Statistics (IBGE) in partnership with the Brazilian Ministry of Health.^14^ The research was planned to take place every three years since 2009, being carried out in public and private schools in Brazil.

PeNSE 2019 data collection took place between April and September, covering Brazilian students aged 13 to 17 years, enrolled and attending the 6th to 9th grades of Elementary School and the 1st to 3rd grades of High School. The following geographical levels were considered: Brazil, Major Regions, Federative Units, Capital Cities, and Federal District.

The PeNSE sample is carried out by clusters in two stages, of which schools correspond to the first selection stage, and classes of enrolled students to the second. In the selected classes, all students were invited to answer the research questionnaire. Sampling weights were calculated considering: the weights of schools, classes and students, adjusted based on data from the 2019 School Census. The PeNSE sample was designed to estimate population parameters for adolescents aged 13 to 17 years, aiming to estimate a proportion (or prevalence) of around 0.5 (50%), with a 4% coefficient of variation^14^.

Students answered a structured, self-administered questionnaire via smartphones, covering information on: socioeconomic status, family context, experimentation and use of cigarettes, alcohol, and other drugs, violence, safety, accidents, and other living conditions^14^. The sample consisted of 125,123 students from 6,612 classes in 4,242 public and private schools, with a loss of 15.2%, with an estimated number of 11,851,941 students aged 13 to 17 years attending school in the country.

### Study variables

"Bullying practice" was the outcome variable, obtained through the question: "In the last 30 days, have you mocked, ridiculed, teased, intimidated, or bullied any of your schoolmates to the point that they felt hurt, upset, offended, or humiliated?" With the response options dichotomized into yes/no.

The independent variables analyzed were:

- *Sociodemographic characteristics*:
  - sex (boys and girls);
  - age (13-15 years and 16-17 years);
  - race/skin color (white, Black, brown, Asian and Indigenous);
  - schools (public or private);
  - mother's level of education (illiterate, some/complete elementary school, some/complete high school, some/complete higher education).
- *Family context*:
  - Living with their mother and/or father — categorized as no (living without their mother and father) or yes (students who live with their mother and/or father);
  - Family supervision — categorized as no (never, rarely, sometimes) or yes (most of the time, parents or guardians always really knew what the teenager was doing);
  - Skipping classes without authorization — categorized as no (never) or yes (one or more times in the last 30 days); being physically assaulted by a family member — categorized as no (none in the last 12 months) or yes (one or more times).
- *Mental health*:
  - Feeling lonely — categorized as no (never, sometimes in the last 12 months) or yes (most of the time, always in the last 12 months);
  - Feeling sad — categorized as no (never, rarely, sometimes) or yes (most of the time and always);
  - Friends — categorized as no (none) or yes (one, two, three, or more friends);
  - Feeling that life is not worth living — categorized as no (never, rarely, sometimes) or yes (most of the time and always).
- *Risk behaviors:*
  Regular use (in the last 30 days) of the following substances:
  - Cigarette;
  - Tobacco;
  - Alcohol;
  - Illicit drugs — whose answers were dichotomized into yes or no. In this domain, the following variable was also included:
  - Sexual initiation, also dichotomized into yes or no.

### Statistical analysis

Initially, the prevalence of bullying was calculated according to: sociodemographic characteristics, family context, mental health, and risk behaviors. To investigate factors associated with reporting bullying practices, a bivariate and multivariate analysis was performed, estimating the crude prevalence ratio (cPR) with their respective 95% confidence intervals (95%CI). The Poisson Regression model with robust variance was used^15^, inserting variables based on the literature, and p<0.05 in the bivariate analysis. To remove variables from the model, the backward technique was used, with statistically significant variables with p<0.05 remaining in the final adjusted model (prevalence ratio - PR). For all analyses, the sampling structure and weights were considered to obtain population estimates. Data were analyzed using the Stata statistical package, version 14.2, suitable for analyzing data obtained from a complex sampling plan.

### Ethical aspects

PeNSE complies with the Guidelines and Regulatory Standards for Research Involving Human Beings and was approved by the National Commission of Ethics in Research of the Ministry of Health (*Comissão Nacional de Ética em Pesquisa* - CONEP/MS), under opinion No. 3,249,268 dated 04/08/2019. Students were informed about the research, their free participation, and the possibility of interrupting it if they did not feel comfortable answering the questions.

## RESULTS

Of the total sample (n=125,123), 50.7% (95%CI 49.9-51.4) were girls, with the majority aged between 13 and 15 years (64.7%; 95%CI 63.2-66.1), and from public schools (85.5%; 95%CI 85.2-85.9). Most had brown skin color (43.2%; 95%CI 42.5-43.9) and their mothers had some/complete high school (27.0%; 95%CI 26.3-27.7), data not shown.

We analyzed data on 19,363 students who bullied schoolmates (12.1%; 95%CI 11.7-12.6). Bullying was more frequent among boys (14.7%; 95%CI 14.1-15.4), with self-declared Black skin color (15.1%; 95%CI 14-16.4), from private schools (13.5%; 95%CI 12.9-14.1%), with no relation to the mother's level of education (Table 1).

**Table 1:** Prevalence of bullying among schoolchildren aged 13 to 17 years according to sociodemographic, family context, mental health, and risk behavior variables. PeNSE 2019.

Variable		n	Bullying practice		
			%	95%CI	
				Lower	Higher
Total		19,636	12.1	11.7	12.6
Sociodemographic characteristics					
Sex					
	Boys	11,675	14.7	14.1	15.4
	Girls	7,625	9.6	9.1	10.1
Age (years)					
	13 to 15	10,596	12.3	11.8	12.8
	16 to 17	5,047	11.8	11	12.6
Race/skin color					
	White	6,828	11.2	10.6	11.9
	Black	2,505	15.1	14	16.4
	Asian	724	11.7	10	13.5
	Brown	8,195	11.9	11.2	12.5
	Indigenous	631	12.41	10.4	14.8
Type of school					
	Public	9,835	11.9	11.4	12.4
	Private	9,528	13.5	12.9	14.1
Mother's level of education					
	Illiterate	671	13.4	11.7	15.3
	Elementary school (some/complete)	3,083	11.7	10.9	12.6
	High school (some/complete)	4,701	11.8	11	12.6
	Higher education (some/complete)	7,663	13.1	12.3	13.9
Family context					
Lives with their mother/father					
	No	1,352	13.9	12.5	15.4
	Yes	17,990	12	11.5	12.4
Family supervision					
	No	8,127	18	17	19
	Yes	11,167	9.6	9.2	10
Skips classes					
	No	14,492	10.7	10.2	11.1
	Yes	4,819	18.1	17.1	19.1
Is physically assaulted by a family member					
	No	12,139	9.7	9.3	10.2
	Yes	6,987	20.7	19.7	21.7
Mental health					
Feels lonely					
	No	7,602	10.1	9.5	10.6
	Yes	11,705	13.8	13.2	14.5
Feels sad					
	No	5,681	10.5	9.9	11.2
	Yes	13,628	12.9	12.4	13.5
Friends					
	One or more	18,509	11.9	11.5	12.4
	Has no friends	826	16.1	14.1	18.3
Life is not worth living					
	No	10,531	10	9.5	10.5
	Yes	8,743	15.5	14.8	16.3
Risk behaviors					
Regular use of cigarette					
	No	17,197	11	10.6	11.4
	Yes	2,144	27.2	25.2	29.2
Regular use of tobacco					
	No	15,301	10.2	9.8	10.7
	Yes	4,059	22.9	21.5	24.3
Regular use of alcohol					
	No	12,754	9.6	9.2	10
	Yes	6,583	18.5	17.6	19.4
Regular use of drugs					
	No	17,592	11.3	10.8	11.7
	Yes	1,759	27.7	25.3	30.1
Sexual intercourse					
	No	11,743	9.6	9.2	10.1
	Yes	7,547	16.6	15.8	17.5

n=19,363. CI: confidence interval.

In the family context, the practice of bullying was higher among those who reported being physically assaulted by family members (20.7%; 95%CI 19.7-21.7), did not live with their mother and/or father (13.7%; 95%CI 10.9-12.6), and skipped classes without informing their family (18.1%; 95%CI 17.1-19.1). Conversely, adolescents who reported family supervision bullied less (9.6%; 95%CI 9.2-10.0) compared to those who denied such supervision.

Regarding mental health characteristics, bullying was more frequent among those who reported sadness (12.9%; 95%CI 12.4-13.5), felling lonely (13.8%; 95%CI 13.2-14.5), had no friends (16.1%; 95%CI 14.1-18.3), and that life was not worth living (15.5%; 95%CI 14.8-16.3).

As for risk behaviors, bullying was more frequent among students who smoked cigarettes (27.2%; 95%CI 25.2-29.2), used tobacco (22.9%; 95%CI 21.5-24.3), alcohol (18.5%; 95%CI 17.6-19.4), and illicit drugs in the last 30 days (27.7%; 95%CI 25.3-30.1) as well as among students who reported sexual initiation (16.6%; 95%CI 15.8-17.5) (Table 1).

In the final model, regarding sociodemographic characteristics, the following were positively associated with the practice of bullying: male adolescents (PR 1.66; 95%CI 1.55-1.77), of self-declared Black (PR 1.23; 95%CI 1.11-1.36) and brown (PR 1.1; 95%CI 1.02-1.18) race/skin color, from private schools (PR 1.29; 95%CI 1.21-1.37). We observed protective factors against bullying among those aged 16 to 17 years (PR 0.82; 95%CI 0.76-0.89) and who had family supervision (PR 0.70; 95%CI 0.66-0.75) (Table 2).

**Table 2:** Risk factors associated with bullying practices among schoolchildren aged 13 to 17 years, PeNSE 2019.

Variable		Univariate model				Multivariate model			
		cPR	95%CI		p	PR	95%CI		p-value
			Lower	Higher			Lower	Higher	
Sociodemographic characteristics									
Sex									
	Boys	1				1.7	1.55	1.77	**<0.001**
	Girls	1.5	1.44	1.64	**<0.001**	1			
Age (years)									
	13 to 15	1				1			
	16 to 17	1	0.89	1.04	0.282	0.8	0.76	0.89	**<0.001**
Race/skin color									
	White	1				1			
	Black	1.4	1.22	1.49	**<0.001**	1.2	1.11	1.36	**<0.001**
	Asian	1	0.88	1.22	0.654	1	0.85	1.18	0.946
	Brown	1.1	0.99	1.13	0.122	1.1	1.02	1.18	0.012
	Indigenous	1.1	0.92	1.33	0.278	1.1	0.9	1.29	0.419
Type of school									
	Public	1				1			
	Private	1.1	1.07	1.21	**<0.001**	1.3	1.21	1.37	**<0.001**
Family context									
Family supervision									
	No	1				1			
	Yes	0.5	0.5	0.57	**<0.001**	0.7	0.66	0.75	**<0.001**
Skips classes									
	No	1				1			
	Yes	1.7	1.6	1.81	**<0.001**	1.2	1.15	1.31	**<0.001**
Is physically assaulted by a family member									
	No	1				1			
	Yes	2.1	2	2.27	**<0.001**	1.7	1.55	1.79	**<0.001**
Mental health									
Feels lonely									
	No	1				1			
	Yes	1.4	1.29	1.46	**<0.001**	1.2	1.09	1.26	**<0.001**
Life is not worth living									
	No	1				1			
	Yes	1.6	1.46	1.65	**<0.001**	1.3	1.19	1.39	**<0.001**
Risk behaviors									
Regular use of tobacco									
	No	1				1			
	Yes	2.2	2.1	2.38	**<0.001**	1.3	1.22	1.47	**<0.001**
Regular use of alcohol									
	No	1				1			
	Yes	1.9	1.82	2.04	**<0.001**	1.4	1.28	1.5	**<0.001**
Regular use of drugs									
	No	1				1			
	Yes	2.5	2.25	2.69	**<0.001**	1.2	1.04	1.31	**0.009**
Sexual intercourse									
	No	1				1			
	Yes	1.7	1.62	1.84	**<0.001**	1.3	1.18	1.35	**<0.001**

cPR: crude prevalence ratio; CI: confidence interval; PR: prevalence ratio. Numbers in bold highlight statistically significant associations (p≤0.05).

In the family context, those who skipped classes without authorization (PR 1.23; 95%CI 1.15-1.31) and who were physically assaulted by family members (PR 1.67; 95%CI 1.55-1.79) were positively associated with bullying. Regarding mental health, adolescents who felt lonely (PR 1.17; 95%CI 1.09-1.26) and reported that life was not worth living (PR 1.28; 95%CI 1.19-1.39) had higher PR for bullying. Regarding risk behaviors, we found positive associations with bullying among those who regularly used tobacco (PR 1.34; 95%CI 1.22-1.47), alcohol (PR 1.38; 95%CI 1.28-1.50), and illicit drugs (PR 1.17; 95%CI 1.04-1.31) and who reported having initiated sexual intercourse (PR 1.26; 95%CI 1.18-1.35) (Table 2).

## DISCUSSION

Around 12% of Brazilian schoolchildren reported bullying their schoolmates. This behavior was associated with boys, aged between 13 and 15 years, of self-declared Black and brown race/skin color, in addition to those enrolled in private schools. In the family context, bullying was associated with students who were physically assaulted by family members, did not live with their mother and/or father, and skipped classes without informing their family. Conversely, family supervision was associated with a lower prevalence ratio of reporting this practice. In terms of mental health, feeling lonely and believing that life is not worth living were associated with a higher prevalence of bullying, which was also higher among those who displayed risk behaviors such as the use of tobacco, alcohol, illicit drugs, and sexual activity.

The prevalence of bullying decreased in 2019 (12.1%; 95%CI 11.6-12.5) compared to 2015 (20.4%; 95%CI 19.2-21.5), probably due to greater awareness and visibility on the subject in school spaces throughout the country^16^. However, the persistence of this practice among schoolchildren is worrisome due to the repercussions on the health and well-being of those involved.

Authors of different national and international studies have also showed that boys or students who identify as male are more likely to bully than girls^8,12^. Researchers analyzed data from 37 European countries and demonstrated that, overall, boys tended to have higher rates of bullying and cyberbullying^17^, when this practice occurs in a virtual environment^3^. The issue of differences between sexes or genders is not yet fully explained by studies, but it is inferred that social and cultural issues are related to the way in which femininity and masculinity are experienced/constructed by adolescents^18^.

The age group of 16 to 17 years was considered a protective factor in this study, in accordance with previous findings^19^. Younger adolescents are likely to bully peers in an attempt to gain acceptance into the group, which is more common during puberty. After this stage, young people may develop greater social awareness and internalize norms against bullying, which may explain a more pronounced decline in bullying among older adolescents^19^.

The greater possibility of involvement of Black/brown race/skin color students as perpetrators of bullying has already been highlighted previously, and may be related to the issue of racial discrimination^20,21^. Researchers have investigated the possible connection between racial discrimination and aggressive behavior^22,23^. Teenagers who are consistently bullied at school because of their race may suffer from mental health problems, which in turn are associated with bullying^24^.

The positive association of bullying among students in private schools should be analyzed with caution. This result demonstrates that this is a phenomenon that goes beyond socioeconomic differences, as previously presented in other editions of PeNSE^25^ or in more local research in Brazil^26^. It is worth noting that there are few data from private schools and they are difficult to access. Nonetheless, we can ponder that the documented differences highlight how socioeconomic issues — assumed based on the type of administrative affiliation of the schools — are relevant to analyzing the dynamics and occurrence of bullying^13,27^.

The association between bullying and the family context is important to understand nuances related to the manifestation of aggressive behavior by adolescents. Family aggression associated with bullying perpetration corroborates previous findings^28,29^. These results are consistent with the theory of intergenerational transmission of violence, which suggests that exposure to family violence increases the likelihood of adolescents engaging in aggressive behaviors^30^.

Regarding the mental health aspects identified, specifically the feeling that "life is not worth living," the association between bullying (suffered and perpetrated) and signs of sadness was evidenced among Norwegian adolescents — victims or aggressors^31^. This suggests that both groups experience so much pressure and stress that the situation ends up resulting in psychological harm^31^. Depression, low self-esteem, suicidal ideation, and attempted suicide were highlighted as the main damages to the mental health of the adolescents involved, which may impact morbidity and mortality among young people^32^. This feeling declared by the participants is related to the psychological construct of hopelessness. Hopelessness is a subjective state with a negative outlook to the future, including feelings of loss of control, confidence in oneself and others, courage and energy to achieve goals^33^. Therefore, we can infer that bullying can also compromise subjective well-being and aggravate episodes of violence, deserving increasing attention in scientific analysis and interventions^5^.

The greatest possibilities of using tobacco, alcohol, and illicit drugs^34^ and sexual initiation^35^ among adolescents who reported bullying have been previously evidenced. Teenagers may engage in bullying behaviors to gain social acceptance^34^, which can also culminate in the adoption of other risk behaviors such as early sexual intercourse and substance use. These can also reduce inhibitory control, increasing aggressive and impulsive behavior. Furthermore, adolescents who bully often face emotional problems and may use substances to self-medicate^36^.

The practice of bullying can therefore be considered a public health issue that has great potential for prevention in the health sector, especially when there is intersectorality in combating violence in schools. In Brazil, the School Health Program (*Programa Saúde na Escola* - PSE) stands out as an intersectoral initiative of the Ministries of Health and Education^37^. Since 2007, the PSE has contributed with several actions on topics relevant to children and adolescents, including the prevention of violence and unhealthy lifestyle habits, the promotion of a culture of peace, and sexual and reproductive health. These activities take place in partnership with the Family Health Strategy and highlight Primary Health Care as a protagonist in guiding children and adolescents^38^, together with the school community. Considering our results, it is worth highlighting the need to strengthen and intensify the PSE as a public policy to improve the quality of life of students and access to health services as well as to contribute to the reduction of social inequalities in the country^39^.

Among the study limitations, we emphasize that the data were collected through a self-report survey, a methodology widely used in this field and to investigating bullying situations. This approach ensures comparability with other similar studies, but it can also introduce biases — such as socially desirable responses and variations in the interpretation of the behavior under analysis. Future studies may be developed to resolve or better explore the presented results, proposing points to overcome these limitations. Another aspect worth mentioning concerns the exclusion of adolescents outside the school environment, who may be more vulnerable to the studied variables. However, research in schools is recommended by the World Health Organization and is frequently carried out by several countries^39^, considering that schools represent a privileged place for actions in the field of health, as they are home to the majority of adolescents^40^.

Conversely, the study strengths include the sample size with national representation of Brazilian students, the standardized data collection method, and the large number of respondents. Our findings can be used to think about strategies to prevent aggressive behavior among students, especially when identifying associated risk factors, such as feelings of loneliness, lack of family supervision, and risk behaviors such as substance use. Based on these findings, interventions can be targeted to address these specific factors, promoting a safer and more welcoming school environment that discourages bullying.

Approximately 12% of Brazilian schoolchildren reported bullying. The associated factors span the sociodemographic, family, mental health, and risk behavior domains. Bullying was associated with boys, of self-declared Black and brown skin color, younger, from private schools, who were physically assaulted by family members, did not live with their parents, or skipped classes without authorization. This behavior was also associated with those who felt lonely, believed that life was not worth living, used substances, and had initiated sexual activity. Family supervision was considered a protective factor. The results mainly point to the importance of considering the influence of the family context, which can be harmful or protective, in the adoption of aggressive behaviors such as bullying. It is worth paying close attention to mental health symptoms that may indicate suffering reflected in the perpetuation of hostile attitudes.
