# Supplementary material for: Bullying practices by students aged 13 to 17 years according to the National Survey of School Health (2019)
Source: Rev Bras Epidemiol. 2025 Feb 10;28:e250003. doi: 10.1590/1980-549720250003 (PMC11809265; doi:10.1590/1980-549720250003)
Supplement: Translation

# Translation: Prática de *bullying* por estudantes de 13 a 17 anos segundo a Pesquisa Nacional de Saúde do Escolar (2019)

**DOI:** 10.1590/1980-549720250003.2

**Authors:** Deborah Carvalho Malta, Juliana Bottoni de Souza, Évelin Angélica Herculano de Morais, Flora Vitória Serena Oliveira Baldi, Flávia Carvalho Malta de Mello, Alexandra Dias Moreira, Wanderlei Abadio de Oliveira

**Affiliations:** I Universidade Federal de Minas Gerais, Escola de Enfermagem, Departamento Enfermagem Materno-infantil e Saúde Pública - Belo Horizonte (MG), Brasil.; II Universidade Federal de Minas Gerais, Escola de Enfermagem - Belo Horizonte (MG), Brasil.; III Universidade de São Paulo, Escola de Enfermagem de Ribeirão Preto - Ribeirão Preto (SP), Brasil.; IV Pontifícia Universidade Católica de Campinas - Campinas (SP), Brasil.

**Keywords:** Bullying, Adolescente, Tabaco, Comportamentos de risco à saúde, Consumo de álcool por menores

## Abstract

**Objetivo:**

Analisar a prevalência de prática de *bullying* e fatores associados entre adolescentes brasileiros, segundo a Pesquisa Nacional de Saúde do Escolar 2019.

**Métodos:**

Estudo transversal, com amostragem por conglomerados. A variável desfecho, prática de *bullying*, foi obtida pela pergunta "Nos últimos 30 dias, você esculachou, zombou, mangou, intimidou ou caçoou algum de seus colegas da escola tanto que ele ficou magoado, aborrecido, ofendido ou humilhado?" (sim/não). Foram analisadas associações com características sociodemográficas, familiares, saúde mental e comportamentos de risco, utilizando regressão de Poisson com variância robusta.

**Resultados:**

12,1% (IC95% 11,7-12,6) dos adolescentes relataram praticar *bullying*. Houve associação positiva entre meninos (RP 1,66; IC95% 1,55-1,77); autodeclarados pretos (RP 1,23; IC95% 1,11-1,36) e pardos (RP 1,1; IC95% 1,02-1,18); de escola privada (RP 1,29; IC95% 1,21-1,37), que sentiam-se solitários (RP 1,17; IC95% 1,09-1,26), que a vida não vale a pena (RP 1,28; IC95% 1,19-1,39), apanharam de familiar (RP 1,67; IC95% 1,55-1,79), faltaram às aulas (RP 1,23; IC95% 1,15-1,31), usavam tabaco (RP 1,34; IC95% 1,22-1,47), álcool (RP 1,38; IC95% 1,28-1,50) e drogas regularmente (RP 1,17; IC95% 1,04-1,31) e tiveram relação sexual (RP 1,26; IC95% 1,18-1,35). A idade 16 e 17 anos (RP 0,82; IC95% 0,76-0,89) e supervisão familiar foram protetoras (RP 0,70; IC95% 0,66-0,75).

**Conclusão:**

Praticar *bullying* foi mais provável entre meninos, mais jovens, com problemas familiares e de saúde mental, e comportamentos de risco. Observa-se a importância de práticas como supervisão familiar contra o *bullying*.

## INTRODUÇÃO

O *bullying* é um grave problema que afeta a saúde e o desenvolvimento de adolescentes em idade escolar^1,2^. Este fenômeno é definido como um tipo de violência que ocorre de maneira repetitiva, baseada em relações desiguais de poder entre vítimas e agressores, de forma intencional. Tradicionalmente, as agressões acontecem nas escolas e podem ser físicas, verbais ou psicológicas, resultando em consequências para todos os envolvidos, sejam vítimas, agressores ou observadores^3^.

Contudo, observa-se que ainda existem poucos estudos que particularizem a experiência dos estudantes identificados como agressores^3,4^. Estudos mostram que os escolares que praticam *bullying* também estão sujeitos a apresentar problemas de saúde mental, baixa capacidade de empatia, necessidade de aceitação no grupo de pares e envolvimento com outras situações de violência^5-7^.

Além disso, foi observado que os meninos praticaram mais todos os tipos de *bullying* (físico, verbal e social) em comparação com as meninas^8^. No que se refere às famílias desse grupo de estudantes, os adolescentes que vivem em contextos de conflito, com altos níveis de comunicação negativa ou com pais autoritários, têm maior chance de apresentarem comportamentos de *bullying* na escola^9,10^. Ser um agressor já esteve, também, associado à baixa escolaridade materna^11,12^ e a condições sociodemográficas desfavoráveis^13^.

Esse cenário revela a complexidade da prática do *bullying* por adolescentes e sinaliza a importância de considerar as particularidades do papel desempenhado pelos estudantes nessas situações. Desta forma, torna-se possível e importante analisar o cenário da prática do *bullying* considerando fatores individuais e contextuais associados a essa prática. Assim, o objetivo deste estudo foi analisar a prevalência da prática de *bullying* e fatores associados entre adolescentes brasileiros, segundo dados da Pesquisa Nacional de Saúde do Escolar (PeNSE) 2019.

## MÉTODOS

Trata-se de um estudo epidemiológico transversal e analítico, que utilizou dados da PeNSE 2019, inquérito nacional realizado pelo Instituto Brasileiro de Geografia e Estatística (IBGE) em parceria com o Ministério da Saúde^14^. A pesquisa foi planejada para ocorrer com periodicidade trienal desde 2009, sendo realizada em escolas públicas e privadas do Brasil.

A coleta de dados da PeNSE 2019 ocorreu entre abril e setembro, abrangendo estudantes brasileiros de 13 a 17 anos, matriculados e frequentando, do 6° ao 9° ano do Ensino Fundamental e da 1° a 3° série do Ensino Médio. Foram considerados os seguintes níveis geográficos: Brasil, Grandes Regiões, Unidades da Federação, Municípios das Capitais e Distrito Federal.

A amostra da PeNSE é realizada por conglomerados em dois estágios, dos quais as escolas correspondem ao primeiro estágio de seleção, e as turmas dos alunos matriculados ao segundo. Nas turmas selecionadas, todos os alunos foram convidados a responder o questionário da pesquisa. Foram calculados pesos amostrais considerando: os pesos das escolas, turmas e alunos, ajustados com base nos dados do Censo Escolar 2019. A amostra da PeNSE foi dimensionada para estimar parâmetros populacionais para os adolescentes de 13 a 17 anos de idade, visando estimar uma proporção (ou prevalência) da ordem de 0,5 (50%), com um coeficiente de variação de 4%^14^.

Os alunos responderam a um questionário estruturado e autoaplicável por meio de *smartphones*, contemplando informações sobre: situação socioeconômica, contexto familiar, experimentação e uso de cigarro, álcool e outras drogas, violência, segurança, acidentes e outras condições de vida^14^. A amostra foi constituída por 125.123 escolares de 6.612 turmas em 4.242 escolas públicas e privadas, com uma perda de 15,2%, estimando-se em 11.851.941 o número de estudantes de 13 a 17 anos frequentando a escola no país.

### Variáveis do estudo

A variável desfecho foi "prática de *bullying*", obtida por meio da pergunta: "Nos últimos 30 dias, você esculachou, zombou, mangou, intimidou ou caçoou algum de seus colegas da escola tanto que ele ficou magoado, aborrecido, ofendido ou humilhado?" Com as opções de resposta dicotomizadas em sim/não.

As variáveis independentes analisadas foram:

- *Características sociodemográficas*:
  - sexo (masculino e feminino);
  - idade (13-15 anos e 16-17 anos); e
  - raça/cor da pele (branca, preta, parda, amarela e indígena),
  - escolas (pública ou privada),
  - escolaridade da mãe (sem escolaridade, ensino fundamental - incompleto/completo, ensino médio - incompleto/completo, ensino superior incompleto/completo).
- *Contexto familiar*:
  - Morar com mãe e/ou pai - categorizada em não (residir sem pai e mãe) ou sim (escolares que residem com pai e/ou mãe);
  - Supervisão familiar - categorizada em não (nunca, raramente, às vezes) ou sim (na maior parte do tempo, sempre pais ou responsáveis sabiam realmente o que o adolescente estava fazendo);
  - Faltar às aulas sem autorização - categorizada em não (nunca) ou sim (1 ou mais vezes nos últimos 30 dias); apanhar de familiar - categorizada em não (nenhuma vez nos últimos 12 meses) ou sim (1 ou mais vezes).
- *Saúde mental*:
  - Sentir-se sozinho/solitário - categorizada em não (nunca, às vezes nos últimos 12 meses) ou sim (na maioria das vezes, sempre nos últimos 12 meses);
  - Sentir-se triste - categorizada em não (nunca, raramente, às vezes) ou sim (na maioria das vezes e sempre)
  - Amigos - categorizada como não (nenhum) ou sim (1, 2, 3, ou mais amigos);
  - Sentir que a vida não vale a pena - categorizada em não (nunca, raramente, às vezes) ou sim (na maioria das vezes e sempre).
- *Comportamentos de risco:*
  Uso regular (nos últimos 30 dias) das seguintes substâncias:
  - Cigarro;
  - Tabaco;
  - Álcool;
  - Drogas ilícitas - cujas respostas foram dicotomizadas em sim ou não. Neste domínio também foi incluída a variável
  - Iniciação sexual, também dicotomizada em sim ou não.

### Análises estatísticas

Inicialmente, realizou-se o cálculo da prevalência de prática de *bullying* segundo: características sociodemográficas, contexto familiar, saúde mental e comportamentos de risco. Para explorar fatores associados com o relato de praticar *bullying,* procedeu-se uma análise bivariada e multivariada, calculando-se a razão de prevalência bruta (RPb) com seus respectivos intervalos de confiança de 95% (IC95%). Foi utilizado o modelo de Regressão de *Poisson* com variância robusta^15^, inserindo-se as variáveis com base na literatura, e p<0,05 na análise bivariada. Para a retirada das variáveis do modelo, utilizou-se a técnica *backward*, permanecendo no modelo final ajustado (RP) as variáveis estatisticamente significativas com p<0,05. Para todas as análises, foram consideradas a estrutura amostral e os pesos para a obtenção de estimativas populacionais. Os dados foram analisados com auxílio do pacote estatístico *Stata*, versão 14.2, adequado para análises de dados obtidos por meio de plano amostral complexo.

### Aspectos éticos

A PeNSE está em conformidade com as Diretrizes e Normas Regulamentadoras de Pesquisa Envolvendo Seres Humanos e foi aprovada pela Comissão Nacional de Ética em Pesquisa do Ministério da Saúde (CONEP/MS), sob parecer n° 3.249.268 de 08/04/2019. Os estudantes foram informados sobre a pesquisa, sua livre participação e sobre a possibilidade de interrompê-la caso não se sentissem à vontade para responder às perguntas.

## RESULTADOS

Do total da amostra (n=125.123), 50,7% (IC95% 49,9-51,4) era do sexo feminino, com a maioria entre 13 e 15 anos (64,7%; IC95% 63,2-66,1) e de escola pública (85,5%; IC95% 85,2-85,9). A maior parte era de cor da pele parda (43,2%; IC95% 42,5-43,9) e cujas mães tinham o ensino médio completo/incompleto (27,0%; IC95% 26,3-27,7), dados não apresentados.

Foram analisados os dados referentes a 19.363 estudantes que praticaram *bullying* contra colegas de escola, (12,1%; IC95% 11,7-12,6). A prática de *bullying* foi mais frequente entre meninos (14,7% IC95% 14,1-15,4), autodeclarados pretos (15,1%; IC95% 14-16,4) de escola privada (13,5%; IC95% 12,9-14,1%), sem relação com a escolaridade da mãe (Tabela 1).

**Tabela 1:** Prevalência da ocorrência da prática de *bullying* entre escolares de 13 a 17 anos segundo variáveis sociodemográficas, do contexto familiar, de saúde mental e comportamentos de risco. PENSE 2019.

Variável		n	Praticar bullying		
			%	IC95%	
				Inferior	Superior
Total		19.636	12,1	11,7	12,6
Características sociodemográficas					
Sexo					
	Masculino	11.675	14,7	14,1	15,4
	Feminino	7.625	9,6	9,1	10,1
Idade (anos)					
	13 a 15	10.596	12,3	11,8	12,8
	16 e 17	5.047	11,8	11	12,6
Raça/cor da pele					
	Branca	6.828	11,2	10,6	11,9
	Preta	2.505	15,1	14	16,4
	Amarela	724	11,7	10	13,5
	Parda	8.195	11,9	11,2	12,5
	Indígena	631	12,41	10,4	14,8
Escola					
	Pública	9.835	11,9	11,4	12,4
	Privada	9.528	13,5	12,9	14,1
Escolaridade da mãe					
	Sem escolaridade	671	13,4	11,7	15,3
	Ensino fundamental (incompleto/completo)	3.083	11,7	10,9	12,6
	Ensino médio (incompleto/completo)	4.701	11,8	11	12,6
	Ensino superior (incompleto/completo)	7.663	13,1	12,3	13,9
Contexto familiar					
Mora com mãe e ou pai					
	Não	1.352	13,9	12,5	15,4
	Sim	17.990	12	11,5	12,4
Supervisão familiar					
	Não	8.127	18	17	19
	Sim	11.167	9,6	9,2	10
Faltar às aulas					
	Não	14.492	10,7	10,2	11,1
	Sim	4.819	18,1	17,1	19,1
Apanhar de familiar					
	Não	12.139	9,7	9,3	10,2
	Sim	6.987	20,7	19,7	21,7
Saúde mental					
Sentir-se solitário					
	Não	7.602	10,1	9,5	10,6
	Sim	11.705	13,8	13,2	14,5
Sentir-se triste					
	Não	5.681	10,5	9,9	11,2
	Sim	13.628	12,9	12,4	13,5
Amigos					
	Um ou mais	18.509	11,9	11,5	12,4
	Não tenho	826	16,1	14,1	18,3
Vida não vale a pena					
	Não	10.531	10	9,5	10,5
	Sim	8.743	15,5	14,8	16,3
Comportamentos de risco					
Cigarro regular					
	Não	17.197	11	10,6	11,4
	Sim	2.144	27,2	25,2	29,2
Tabaco regular					
	Não	15.301	10,2	9,8	10,7
	Sim	4.059	22,9	21,5	24,3
Álcool regular					
	Não	12.754	9,6	9,2	10
	Sim	6.583	18,5	17,6	19,4
Drogas regular					
	Não	17.592	11,3	10,8	11,7
	Sim	1.759	27,7	25,3	30,1
Relação sexual					
	Não	11.743	9,6	9,2	10,1
	Sim	7.547	16,6	15,8	17,5

n=19.363. IC: intervalo de confiança.

No contexto familiar, a prática de *bullying* foi maior entre os que relataram apanhar de familiares (20,7%; IC95% 19,7**-**21,7), não moravam com mãe e/ou pai (13,7%; IC95% 10,9-12,6) e faltavam as aulas sem comunicar a família (18,1%; IC95% 17,1**-**19,1). Por outro lado, os adolescentes que relataram supervisão familiar praticaram menos *bullying* (9,6%; IC95% 9,2**-**10,0) em comparação àqueles que negaram essa supervisão.

No que diz respeito às características de saúde mental, a prática de *bullying* foi mais frequente entre os que relataram tristeza (12,9%; IC95% 12,4**-**13,5), sentiam-se solitários (13,8%; IC95% 13,2**-**14,5), não tinham amigos (16,1%; IC95% 14,1**-**18,3) e referiram que a vida não vale a pena (15,5%; IC95% 14,8**-**16,3).

Com relação aos comportamentos de risco, praticar *bullying* foi mais frequente em escolares que fumaram cigarro (27,2%; IC95% 25,2**-**29,2), usaram tabaco (22,9%; IC95% 21,5**-**24,3), álcool (18,5%; IC95% 17,6**-**19,4) e drogas ilícitas nos últimos 30 dias (27,7%; IC95% 25,3**-**30,1), bem como entre os estudantes que relataram iniciação sexual (16,6%; IC95% 15,8**-**17,5) (Tabela 1).

No modelo final, no que tange às características sociodemográficas, foi associado positivamente à prática de *bullying*: adolescentes do sexo masculino (RP 1,66; IC95% 1,55**-**1,77), da raça/cor da pele autodeclarada preta (RP 1,23; IC95% 1,11**-**1,36) e parda (RP 1,1; IC95%1,02**-**1,18), de escolas privadas (RP 1,29; IC95% 1,21**-**1,37). Fatores protetores à prática de *bullying* foram observados entre aqueles que tinham de 16 a 17 anos (RP 0,82 IC95% 0,76**-**0,89) e que tinham supervisão familiar (RP 0,70; IC95% 0,66**-**0,75) (Tabela 2).

**Tabela 2:** Fatores de risco associados a praticar *bullying* entre escolares de 13 a 17 anos, PeNSE 2019.

Variável		Modelo univariado				Modelo multivariado			
		RPb	IC95%		p	RP	IC95%		p-valor
			Inferior	Superior			Inferior	Superior	
Características sociodemográficas									
Sexo									
	Masculino	1				1,7	1,55	1,77	**<0,001**
	Feminino	1,5	1,44	1,64	**<0,001**	1			
Idade (anos)									
	13 a 15	1				1			
	16 e 17	1	0,89	1,04	0,282	0,8	0,76	0,89	**<0,001**
Raça/cor da pele									
	Branca	1				1			
	Preta	1,4	1,22	1,49	**<0,001**	1,2	1,11	1,36	**<0,001**
	Amarela	1	0,88	1,22	0,654	1	0,85	1,18	0,946
	Parda	1,1	0,99	1,13	0,122	1,1	1,02	1,18	0,012
	Indígena	1,1	0,92	1,33	0,278	1,1	0,9	1,29	0,419
Escola									
	Pública	1				1			
	Privada	1,1	1,07	1,21	**<0,001**	1,3	1,21	1,37	**<0,001**
Contexto familiar									
Supervisão familiar									
	Não	1				1			
	Sim	0,5	0,5	0,57	**<0,001**	0,7	0,66	0,75	**<0,001**
Faltar às aulas									
	Não	1				1			
	Sim	1,7	1,6	1,81	**<0,001**	1,2	1,15	1,31	**<0,001**
Apanhar de familiar									
	Não	1				1			
	Sim	2,1	2	2,27	**<0,001**	1,7	1,55	1,79	**<0,001**
Saúde mental									
Sentir-se solitário									
	Não	1				1			
	Sim	1,4	1,29	1,46	**<0,001**	1,2	1,09	1,26	**<0,001**
Vida não vale a pena									
	Não	1				1			
	Sim	1,6	1,46	1,65	**<0,001**	1,3	1,19	1,39	**<0,001**
Comportamentos de risco									
Tabaco regular									
	Não	1				1			
	Sim	2,2	2,1	2,38	**<0,001**	1,3	1,22	1,47	**<0,001**
Álcool regular									
	Não	1				1			
	Sim	1,9	1,82	2,04	**<0,001**	1,4	1,28	1,5	**<0,001**
Drogas regulares									
	Não	1				1			
	Sim	2,5	2,25	2,69	**<0,001**	1,2	1,04	1,31	**0,009**
Relação sexual									
	Não	1				1			
	Sim	1,7	1,62	1,84	**<0,001**	1,3	1,18	1,35	**<0,001**

RPb: razão de prevalência bruta; IC: intervalo de confiança; RP: razão de prevalência. Os números marcados em negrito, são para evidenciar as associações estatisticamente significativas (p≤0,05).

No contexto familiar, foi associado positivamente à prática de *bullying*, aqueles que faltaram às aulas sem permissão (RP 1,23; IC95% 1,15**-**1,31) e apanharam de familiar (RP 1,67; IC95% 1,55**-**1,79). Com relação à saúde mental, os adolescentes que se sentiam solitários (RP 1,17; IC95% 1,09**-**1,26) e referiram que vida não vale a pena (RP 1,28; IC95% 1,19**-**1,39) apresentaram maiores RP de prática de *bullying*. A respeito dos comportamentos de risco, foram observadas associações positivas a praticar *bullying* entre aqueles que usaram tabaco (RP 1,34; IC95% 1,22**-**1,47), álcool (RP 1,38; IC95% 1,28**-**1,50), drogas ilícitas regularmente (RP 1,17; IC95% 1,04**-**1,31) e referiram terem iniciado relação sexual (RP 1,26; IC95% 1,18**-**1,35) (Tabela 2).

## DISCUSSÃO

Cerca de 12% dos escolares brasileiros relataram praticar *bullying* contra seus colegas de escola. Essa conduta foi associada aos meninos, com idades entre 13 e 15 anos, de raça/cor da pele autodeclarada preta e parda, além daqueles matriculados em escolas privadas. No contexto familiar, a prática de *bullying* foi associada aos estudantes que sofriam agressões de familiares, não moravam com mãe e/ou pai e faltavam às aulas sem comunicar a família. Por outro lado, a supervisão familiar foi associada a uma menor razão de prevalência de relato dessa prática. Em termos de saúde mental, sentir solidão e acreditar que a vida não vale a pena foram associados à maior prevalência de prática de *bullying*, que também foi maior entre aqueles que apresentaram comportamentos de risco como o uso de tabaco, álcool, drogas ilícitas e atividade sexual.

Observa-se que a prevalência da prática de *bullying* diminuiu em 2019 (12,1%; IC95% 11,6-12,5) se comparada a 2015 (20,4%; IC95% 19,2-21,5), provavelmente em decorrência da maior conscientização e visibilidade sobre o assunto nos espaços escolares em todo o país^16^. Entretanto, a persistência desta prática entre os escolares é preocupante pelas repercussões na saúde e no bem-estar dos envolvidos.

Diferentes estudos, nacionais e internacionais, também já revelaram que os meninos ou estudantes que se declaram do sexo masculino praticam mais *bullying* que as meninas^8,12^. Uma investigação que analisou dados de 37 países da Europa revelou que, em geral, os meninos tendiam a apresentar taxas mais altas de prática de *bullying* tradicional e *cyberbullying* ^17^, quando esta prática ocorre em ambiente virtual^3^. A questão das diferenças entre os sexos ou gêneros ainda não é totalmente explicada pelos estudos, mas infere-se que as questões sociais e culturais estejam relacionadas ao modo como a feminilidade e a masculinidade são vivenciadas/construídas pelos adolescentes^18^.

A faixa etária de 16 a 17 anos foi considerada fator protetor neste estudo, conforme achados prévios^19^. Provavelmente, os adolescentes mais jovens tendem a intimidar colegas na tentativa de aceitação no grupo, o que ocorre mais na puberdade. Após esta fase, os jovens podem desenvolver maior consciência social e internalizar normas contra a intimidação, o que pode explicar um declínio mais acentuado de *bullying* entre os adolescentes mais velhos^19^.

A maior possibilidade de envolvimento de escolares de raça/cor da pele preta/parda enquanto perpetradores de *bullying* já foi evidenciado anteriormente, podendo estar relacionado à questão de discriminações raciais^20,21^. Pesquisas investigaram a possível conexão entre a discriminação racial e comportamentos agressivos^22,23^. Adolescentes que são constantemente maltratados na escola por causa de sua raça podem sofrer prejuízos na saúde mental, que, por sua vez, estão associados à perpetração de *bullying* ^24^.

A associação positiva de prática de *bullying* entre estudantes de escolas privadas deve ser analisada com cautela. Esse resultado demonstra que se trata de um fenômeno que ultrapassa diferenças socioeconômicas, conforme apresentado anteriormente em outras edições da PeNSE^25^ ou em pesquisas mais locais no Brasil^26^. Nota-se que os dados oriundos de escolas privadas são de difícil acesso e em menor quantidade. Contudo, pode-se refletir que as diferenças documentadas assinalam como as questões socioeconômicas — presumidas com base no tipo de dependência administrativa das escolas — são relevantes para analisar a dinâmica e a ocorrência do *bullying* ^13,27^.

A associação entre praticar *bullying* e o contexto familiar é um indício importante para entender nuances relacionadas à manifestação de comportamentos agressivos por parte dos adolescentes. A agressão familiar associada à perpetração do *bullying* corrobora achados anteriores^28,29^. Esses resultados são consistentes com a teoria da transmissão intergeracional da violência, que sugere que a exposição à violência familiar aumenta a probabilidade do adolescente se envolver em comportamentos agressivos^30^.

No que concerne aos aspectos de saúde mental identificados, especificamente a sensação de que a "vida não vale a pena ser vivida", a associação entre *bullying* (sofrido e perpetrado) e indícios de tristeza foi evidenciada entre adolescentes noruegueses — vítimas ou agressores^31^. Isso sugere que ambos os grupos vivenciam tanta pressão e estresse que a situação acaba resultando em agravos psicológicos^31^. A depressão, baixa autoestima, ideação e tentativas de suicídio foram evidenciadas como principais danos à saúde mental dos adolescentes envolvidos, podendo impactar na morbimortalidade entre os jovens^32^. Essa sensação declarada pelos participantes está relacionada ao construto psicológico de desesperança. A desesperança é um estado subjetivo com uma perspectiva negativa do futuro, incluindo sensações de perda de controle, confiança em si e nos outros, coragem e energia para alcançar objetivos^33^. Infere-se, assim, que a prática de *bullying* também pode comprometer o bem-estar subjetivo e agravar os episódios de violência, merecendo cada vez mais destaque na análise científica e nas intervenções^5^.

As maiores possibilidades de uso de tabaco, álcool e drogas ilícitas^34^ e iniciação sexual^35^ entre adolescentes que relataram prática de *bullying* foram anteriormente evidenciadas. Adolescentes podem se engajar em comportamentos de *bullying* para adquirir aceitação social^34^, o que também pode culminar na adesão a outros comportamentos de risco, como relação sexual precoce e uso de substâncias. Estas podem, ainda, reduzir o controle inibitório, aumentando comportamentos agressivos e impulsivos. Além disso, adolescentes que praticam *bullying* muitas vezes enfrentam problemas emocionais, e podem utilizar substâncias para automedicação^36^.

A prática do *bullying* pode ser considerada, portanto, um problema de saúde pública que possui grande potencial de prevenção no setor da saúde, principalmente quando há intersetorialidade no combate à violência nas escolas. No Brasil, destaca-se o Programa Saúde na Escola (PSE) como uma iniciativa intersetorial dos Ministérios da Saúde e da Educação^37^. Desde 2007, o PSE tem contribuído com diversas ações acerca de temas relevantes para crianças e adolescentes, entre eles a prevenção da violência e de hábitos de vida não saudáveis, a promoção da cultura de paz e a saúde sexual e reprodutiva. Essas atividades acontecem em parceria com a Estratégia Saúde da Família e destacam a Atenção Primária à Saúde como protagonista na orientação de crianças e adolescentes^38^, junto à comunidade escolar. Considerando os resultados do presente estudo, salienta-se a necessidade de fortalecimento e intensificação do PSE como política pública para melhorar a qualidade de vida dos estudantes e o acesso aos serviços de saúde, bem como contribuir para a redução das iniquidades sociais no país^39^.

Entre as limitações do estudo, ressalta-se que os dados foram coletados por meio de inquérito com autorrelato, uma metodologia amplamente utilizada nesse campo e para investigar situações de *bullying*. Essa abordagem assegura a comparabilidade com outros estudos similares, mas também pode introduzir vieses, como respostas socialmente desejáveis e variações na interpretação do comportamento em análise. Estudos futuros poderão ser desenvolvidos para dirimir ou explorar melhor os resultados apresentados, propondo pontos de superação para essas limitações. Outro aspecto a ser mencionado diz respeito à exclusão de adolescentes fora do ambiente escolar, que podem apresentar uma maior vulnerabilidade quanto às variáveis estudadas. Entretanto, pesquisas no âmbito escolar são recomendadas pela Organização Mundial de Saúde e frequentemente realizadas por vários países^39^, uma vez que a escola representa lugar privilegiado para ações no âmbito de saúde, já que concentra a maioria dos adolescentes^40^.

Por outro lado, os pontos fortes do estudo incluem o tamanho amostral com representatividade nacional dos estudantes brasileiros, o método de coleta de dados padronizado e o grande número de respondentes. Os achados revelados poderão ser utilizados para pensar estratégias de prevenção do comportamento agressivo entre estudantes, especialmente ao identificar os fatores de risco associados, como o sentimento de solidão, a falta de supervisão familiar e comportamentos de risco, como o uso de substâncias. Com base nesses resultados, as intervenções podem ser direcionadas para abordar esses fatores específicos, promovendo um ambiente escolar mais seguro e acolhedor que desestimule a prática do *bullying*.

Aproximadamente 12% dos escolares brasileiros relataram prática de *bullying*. Os fatores associados perpassam pelos domínios sociodemográficos, familiares, de saúde mental e de comportamentos de risco. A prática de *bullying* foi associada aos meninos, autodeclarados pretos e pardos, mais jovens, de escolas particulares, que sofriam agressões familiares, não moravam com os pais ou faltavam às aulas sem permissão. Essa conduta também foi associada àqueles que se sentiam solitários, acreditavam que a vida não vale a pena, faziam uso de substâncias e haviam iniciado atividade sexual. A supervisão familiar foi considerada fator de proteção. Os resultados direcionam, principalmente, para a importância de se considerar a influência do contexto familiar, que pode ser nociva ou protetiva, na adoção de conduta agressiva como o *bullying*. Ressalta-se a necessidade do olhar atento às manifestações de saúde mental quem podem indicar sofrimento reverberado na perpetuação de atitudes hostis.
